# Immune Checkpoint Blockade in Melanoma – Earlier is Better?

**DOI:** 10.15388/Amed.2024.31.1.16

**Published:** 2024-02-27

**Authors:** Vincas Urbonas, Audrius Dulskas, Edita Baltruškevičienė, Daiva Dabkevičienė

**Affiliations:** 1 Laboratory of Clinical Oncology, National Cancer Institute, Vilnius, Lithuania; 2 Biobank, National Cancer Institute, Vilnius, Lithuania

**Keywords:** melanoma, neoadjuvant treatment, immunotherapy, Melanoma, neoadjuvantinis gydymas, imuninės patikros slopikliai, imunoterapija

## Abstract

Administering checkpoint inhibition before surgery, known as neoadjuvant therapy, shows promise in treating bulky yet resectable melanomas, and researchers are investigating its potential in various other cancer types. This approach boasts a considerable success in high pathologic response rate, a factor directly linked to survival rates. The routine availability of biopsies presents a distinct chance to comprehend treatment responses. Neoadjuvant ICIs offer advantages like T cell expansion, treatment assessment through surgical specimens, and potential tumor size reduction for better surgical outcomes. However, further research is needed to optimize patient selection and treatment protocols.

## Abstract (it)

Imuninės patikros slopiklių (IPS) skyrimas prieš operaciją, žinomas kaip neoadjuvantinis gydymas, yra perspektyvus metodas gydant lokaliai išplitusias rezektabilias melanomas. Šis gydymo būdas taip pat yra plačiai tyrinėjamas kitų vėžinių susirgimų kontekste. Taikant šį gydymą stebimas ženklus patologinis atsakas, o šis veiksnys tiesiogiai susijęs su paciento išgyvenamumu. Be to, taikant neoadjuvantinį gydymą, išlieka galimybė atlikti pakartotinas biopsijas, kurios savo ruožtu suteikia puikią galimybę įvertinti ir stebėti dinamikoje atsaką į gydymą. Nežiūrint visų šio gydymo teikiamų privalumų, reikalingi tolimesni klinikiniais tyrimais pagrįsti duomenys, kurie padėtų optimizuoti gydymo protokolus bei tinkamų šiam gydymui pacientų parinkimą.

Patients with macroscopic resectable stage III melanoma typically undergo therapeutic lymph node dissection (TLND). However, even with TLND, a significant proportion of patients with high-risk disease will experience recurrence within two years without additional adjuvant therapy [1]. In recent years, the U.S. Food and Drug Administration (FDA) and the European Medicines Agency (EMA) have approved the use of adjuvant therapy for stage III melanoma. This includes the anti-PD-1 antibodies pembrolizumab or nivolumab, and for patients with tumors having BRAF V600 E/K mutations, adjuvant BRAF/MEK inhibition with trametinib/dabrafenib. While these adjuvant therapies have improved recurrence-free survival (RFS), a significant proportion of patients (30–50%) still experience disease recurrence within the first two years [1].

To address this unmet therapeutic need, research efforts in the past years were focused on exploring the use of immune-checkpoint inhibitors (ICIs) as neoadjuvant treatment modalities (refer to the treatment given before surgery) for patients with resectable stage III melanoma. Indeed, data from several clinical trials performed in recent years have shown encouraging efficacy of neoadjuvant immune checkpoint inhibitors (ICIs) in patients with resectable macroscopic stage III melanoma. These clinical trials have provided evidence of the following benefits of neoadjuvant ICI therapy in patients with stage III melanoma [2, 3]:

1. Favorable survival outcomes: neoadjuvant ICI therapy has shown promising results in terms of recurrence-free survival and overall survival in patients with resectable macroscopic stage III melanoma. These findings suggest that neoadjuvant ICIs can help improve long-term outcomes by eliminating micrometastases and enhancing the immune response against residual disease.
2. Assess the effectiveness of treatment on a per-patient basis for potential supplementary adjuvant therapy if necessary.
3. Diminish the tumor load prior to surgical intervention.
4. Utilize data on pathological response as substitute indicators for both relapse-free and overall survival.

Recently in NEJM [4] Patel and colleagues presented data of a randomized phase 2 trial, where patients with clinically detectable, measurable stage IIIB to IVC melanoma (according the AJCC 7th edition), which could be surgically removed, were assigned into two treatment groups. The first group (154 ptients) received 3 doses of neoadjuvant pembrolizumab, followed by surgery, and then 15 doses of adjuvant pembrolizumab. The second group (159 patients) underwent surgery first and then received pembrolizumab as adjuvant therapy for approximately one year or until disease recurrence or unacceptable side effects occurred.

The primary endpoint of the study was event-free survival, which refers to the length of time patients remained free from events such as disease progression or toxic effects that prevented surgery. Events were also defined as the inability to remove all visible disease, surgical complications, toxic effects that delayed the initiation of adjuvant therapy within 84 days after surgery, recurrence of melanoma after surgery, or death from any cause. The intention-to-treat population was analyzed for the primary endpoint, meaning that all patients initially assigned to a treatment group were included, regardless of whether they completed the full treatment or experienced any events. The trial also evaluated the safety, assessing any adverse effects or complications experienced by the patients.

In a landmark analysis, which focuses on the outcomes at a specific time point (in this case, 2 years), the event-free survival rate was found to be 72% (95% confidence interval [CI], 64 to 80) in the neoadjuvant–adjuvant group, whereas it was 49% (95% CI, 41 to 59) in the adjuvant-only group (hazard ratio, 0.58; *P* = .004). This indicates that the neoadjuvant approach, where patients received pembrolizumab prior to surgery and continued with adjuvant therapy, led to a higher percentage of patients remaining free from events at the two years timepoint. Regarding safety, the percentage of patients experiencing treatment-related adverse events of grades 3 or higher during therapy was 12% in the neoadjuvant–adjuvant group and 14% in the adjuvant-only group. This suggests that toxicities were equal in both arms with a similar incidence of significant adverse events in both groups.

These results highlight the potential benefit of incorporating neoadjuvant pembrolizumab as a new treatment modality in daily clinical practice for stage IIIB to IVC melanoma, as it demonstrated improved event-free survival compared to the adjuvant-only approach. Simultaneously, the ongoing NADINA trial (NCT04949113) is actively enrolling participants to explore the comparison between neoadjuvant nivolumab plus ipilimumab versus adjuvant nivolumab in macroscopic stage III melanoma within a phase III framework [5].

Proof of concept for neoadjuvant treatment with ICIs in melanoma patients was demonstrated in previous trials. The OpACIN investigation [6] delved into neoadjuvantICI use in stage III melanoma patients. It compared 4 cycles of adjuvant ipilimumab plus nivolumab with a combination of 2 cycles each of neoadjuvant and adjuvant ipilimumab plus nivolumab. Results highlighted the feasibility of neoadjuvant ipilimumab plus nivolumab, revealing an unexpectedly high pathologic response rate (pRR) of 78% and an increased diversity of tumor-infiltrating T cell clones compared to adjuvant therapy. Despite these positive outcomes, both treatment arms experienced substantial toxicity, with 90% of patients reporting grade 3–4 immunotherapy-related adverse events (irAEs).

The OpACIN-Neo phase II trial explored three different neoadjuvant dosing schedules: arm one involved 2 cycles of ipilimumab 3 mg/kg plus nivolumab 1 mg/kg every 3 weeks, arm two included 2 cycles of ipilimumab 1 mg/kg plus nivolumab 3 mg/kg every 3 weeks, and arm three comprised 2 cycles of ipilimumab 3 mg/kg every 3 weeks followed directly by 2 cycles of nivolumab 3 mg/kg every 2 weeks. In the initial 3-month period, severe (grade 3–4) immune-related adverse events (irAEs) were noticed in 40% of patients in arm one, 20% in arm two, and 50% in arm three. Complete pRR responses were observed in 57% of patients in arm one, 47% in arm two, and 23% in arm three. Ultimately, after considering all three schedules, it was concluded that the optimal neoadjuvant dosing regimen, balancing efficacy and toxicity, consisted of 2 cycles of the combined regimen involving ipilimumab 1 mg/kg and nivolumab 3 mg/kg [7].

In a combined examination of six melanoma neoadjuvant trials conducted by Menzies et al., two trials encompassed BRAF/MEK targeted therapy while four trials involved ICIs [8]. The rates of pathological complete response (pCR) were comparable between targeted therapy and immunotherapy (47% versus 37%, p>0.05); however, the rate was notably higher among patients who underwent combination immunotherapy compared to monotherapy (44% versus 21%, p=0.023). Among those treated with targeted therapy, individuals achieving pCR showed a trend toward better Recurrence-Free Survival (RFS) at 2 years (60%) compared to those with a partial response (PR) (44%). Conversely, patients treated with ICI, whether achieving pCR or PR, exhibited minimal recurrence rates (2-year RFS 100% and 96%, respectively). Additionally, patients subjected to combination immunotherapy demonstrated superior Overall Survival (OS) compared to those receiving monotherapy (2-year OS 96% versus 76%, p=0.006).

Do we have a scientific based rationale for neoadjuvant ICI in patients with melanoma? Yes, we do. The strategy of neoadjuvant immunotherapy in cancer treatment could be based on several key factors [9]:

1. Induction of T cell expansion: immunotherapy, particularly immune checkpoint inhibitors like anti-PD-1 antibodies, can activate and enhance the function of T cells. By administering immunotherapy before surgery, it is possible to induce the expansion and activation of T cells within the tumor microenvironment. This activation can help eliminate cancer cells and initiate an antitumor immune response.
2. Neoadjuvant immunotherapy is particularly valuable in melanoma patients with lower tumour burden, when T cell function is relatively less impaired. At advanced stages, the immune system may be more compromised, and tumor burden and immune evasion mechanisms may be more pronounced. By initiating immunotherapy earlier, there is a higher likelihood of achieving an effective immune response against the tumor.
3. Feasibility of assessing treatment effects: neoadjuvant immunotherapy allows for the routine assessment of treatment effects through biopsy of surgical specimens. By analyzing these specimens, researchers and clinicians can evaluate the extent of immune cell infiltration, changes in tumor characteristics, and biomarkers indicative of treatment response. This real-time assessment provides valuable insights into the effectiveness of the immunotherapy and its impact on the tumor microenvironment. Detection of pathologic complete response may reduce the need for imaging and follow ups, whereas patients with pathologic nonresponse would profit from more intense follow up schedule.
4. Reduction of tumor size and improved surgical outcomes: immunotherapy has the potential to shrink tumors before surgery. By reducing tumor size, neoadjuvant immunotherapy may improve the feasibility and success of surgical resection. Smaller tumor size can facilitate complete resection, potentially reducing the risk of leaving behind residual cancer cells. Additionally, the reduction in tumor burden may lead to improved surgical outcomes and decreased morbidity associated with extensive surgeries.

In summary, neoadjuvant immunotherapy offers the possibility of harnessing the immune system’s antitumor response [9, 10] earlier in the treatment journey (Figure 1), optimizing T cell function, evaluating treatment response, and potentially enhancing surgical outcomes. These factors contribute to the rationale and promise behind this approach in melanoma and other solid tumors treatment. While neoadjuvant therapy holds promise, current ICIs regimens exhibit relatively high toxicity rates. Further investigation is necessary to maintain efficacy while mitigating toxicity, and the identification of predictive biomarkers in this context would be immensely beneficial. It‘s essential to recognize that individual patient responses can differ, necessitating additional research to optimize patient selection, treatment regimens, and long-term outcomes in this setting.

**Figure 1:**
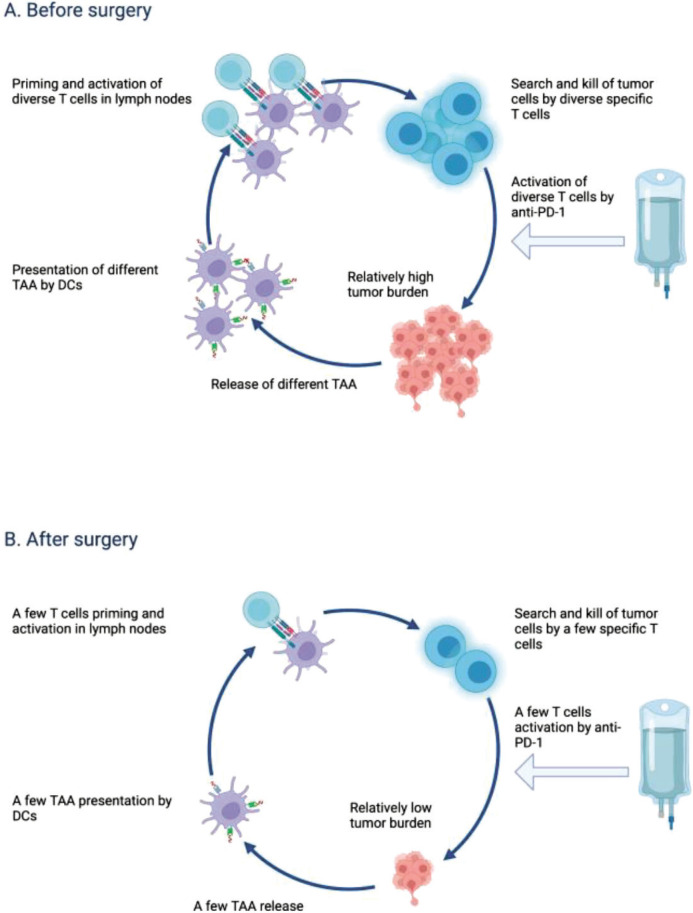
Neoadjuvant and adjuvant treatment impact on cancer-immunity cycle. Diversity and higher amount of specific T cell clones in neoadjuvant treatment setting (A) predict stronger anticancer immune response in comparison with adjuvant treatment (B). Abreviations: DC, dendritic cell; TAA, tumor-associated antigens. Figure created with BioRender (BioRender.com)

## Data Availability

All data provided in the manuscript is available in Pubmed.
